# Low-Level Toxic Metal Exposure in Healthy Weaning-Age Infants: Association with Growth, Dietary Intake, and Iron Deficiency

**DOI:** 10.3390/ijerph14040388

**Published:** 2017-04-06

**Authors:** Jungil Choi, Ju Young Chang, Jeana Hong, Sue Shin, Jeong Su Park, Sohee Oh

**Affiliations:** 1 Department of Electrical Engineering and Computer Science, Seoul National University, Seoul 08826, Korea; gonasa82@snu.ac.kr; 2 Department of Pediatrics, Seoul National University College of Medicine, Seoul 03080, Korea; 3 Department of Pediatrics, Seoul Metropolitan Government Seoul National University Boramae Medical Center, Seoul 07061, Korea; 4 Department of Pediatrics, Kangwon National University School of Medicine, Chuncheon 24289, Korea; dochong@hanmail.net; 5 Department of Laboratory Medicine, Seoul National University College of Medicine, Seoul 03080, Korea; jeannie@snu.ac.kr (S.S.); mdjs0721@naver.com (J.S.P.); 6 Department of Laboratory Medicine, Seoul Metropolitan Government Seoul National University Boramae Medical Center, Seoul 07061, Korea; 7 Department of Medical Statistics, Seoul Metropolitan Government Seoul National University Boramae Medical Center, Seoul 07061, Korea; oh.sohee@gmail.com

**Keywords:** arsenic, cadmium, mercury, lead, infant, breast-feeding, diet, iron deficiency, growth, head circumference

## Abstract

Even low levels of toxic metal exposure (As, Cd, Hg, and Pb) in infancy might be harmful to children’s development. This study investigated toxic metal exposure on healthy weaning-age infants and its relationship with growth, diet, and iron/anemia status. The weight, height, head circumference, whole blood levels of four toxic metals, hemoglobin, and serum ferritin of healthy infants was measured. Among 210 infants with a median age of 11.4 months (interquartile range: 10.5–12.0), the median levels of As, Cd, Hg, and Pb were 1.2 μg/L, 0.05 μg/L, 0.8 μg/L, and 0.83 μg/dL, respectively. In adjusted linear regression models, post-birth weight gain (Pb) and current head circumference (As, Pb) were negatively associated with toxic metal levels. In multiple linear regression or logistic regression analysis, the duration of breastfeeding (all four metals), perceived adequacy of rice-based food intake (As), regular fish intake (As, Hg), and iron deficiency with/without anemia (Cd, Pb) were associated with increased toxic metal levels. Although levels of toxic metals may not usually be high in this population, individual exposure risk may need to be assessed after considering the type of feeding or intake of complementary foods and the iron/anemia status while evaluating growth status during late infancy.

## 1. Introduction

Arsenic, cadmium, mercury, and lead are established or potentially neurotoxic and genotoxic metals that lead to serious health problems in the case of human exposure [1,2,3,4]. Excessive exposure to these toxic metals can irreversibly damage normal infant development [5,6,7,8]. Recently, even low-level toxic metal exposure, unrelated to environmental exposure in high-risk residential areas, has been reported to adversely affect the normal physical growth of fetuses, infants and/or older children [9,10,11,12].

Among the four toxic metals, Pb is the one that is best known to impede children’s growth, as it accumulates in the long bones and leads to bone damage [8,13]. Both prenatal and postnatal low-level Pb exposure (<5.0 µg/dL) has been reported to be inversely associated with post-birth growth of Korean infants and children [12,14,15]. However, few studies have investigated the dose–response association of low-level postnatal Pb exposure and the subsequent postnatal growth of children in late infancy, which is generally regarded as a high-risk period for Pb exposure [16,17]. For As, Cd, or Hg, prenatal low-level exposure has been reported to have unfavorable effects on intrauterine or post-birth growth in some studies [9,10,18,19,20]. However, evidence of this is inconsistent in the current literature [11,13,21]. Moreover, studies on the effects of low-level postnatal As, Cd, or Hg exposure on the postnatal growth of infants or children are scarce as well. In addition, most studies investigated the effect of a single metal; only a few studies have simultaneously evaluated and compared the effect of low levels of multiple toxic metals on the growth of infants or children in the same population [22,23,24]. Factors such as different levels of exposure to toxic metals, differing amounts of materials that counteract or augment the harmful action of the toxic metals, or incomplete adjustment for other growth-affecting factors might contribute to the mixed results among previous studies [11,12,25,26].

The weaning period, also known as the period of complementary feeding (CF), is critical to the physical and mental development of children [27]; this may also be a critical period for the examination of the association between toxic metal exposure and children’s growth and development. During this period, infants are exposed to toxic metals through dietary intake, putting contaminated non-food objects into their mouths, and indirect smoking [17]. Among these, diet may be the most important source of toxic metal exposure among the general population [28]. Both breastmilk and solid food can be important sources of toxic metal exposure during infancy.

All four toxic metals have been reported to be transmitted to babies by breastfeeding, although the degree of excretion of toxic metals in breastmilk seems different for different toxic metals [29]. Formula and its mixing water have also been reported as sources of toxic metal exposure [30,31]. The exposure pattern to toxic metals through the intake of contaminated solid foods may be similar to that of adults living in the same area. In Korea, the main source of complementary food usually includes rice-based food, red meat, fish and vegetables. However, rice can have elevated As levels [32]. Korean rice contained a similar amount of As to that of U.S. rice or Taiwan rice, although the level is distinctively lower than that of Bangladeshi rice [33,34,35]. Rice has been reported as an important source of As exposure among Korean immigrants in the US [36]. Fish intake has also been reported to be associated with increased As exposure in Korean adults and increased Hg exposure in Korean children [25,37]. According to a previous study on Korean children, grains, fish, shellfish, and seaweeds were important sources of Cd exposure; sources of Pb exposure were fruits, vegetables, and grains, rather than meats [38]. As, Cd, and Hg levels were higher among Korean adults than in those reported in western countries in a recent Korean national environmental health survey [39].

In addition, during the weaning period, infants are more likely to lack essential trace elements, which may serve to counteract the effects of toxic minerals [27,40]. For example, iron deficiency is the most common during this period. Childhood iron deficiency causes not only anemia but also retarded physical development [41]. In addition, if iron deficiency occurs during the first year of life, then permanent neural system disorders could arise [42]. Iron has also been reported to be involved in the metabolism of toxic metals, and iron deficiency has been reported to increase the absorption of Cd, Pb, and other toxic metals [43].

Nevertheless, population-based epidemiological studies examining the relationships among diet intake, iron status, and the toxic metal burden in weaning-age infants are limited. In addition, few studies have evaluated blood levels of toxic metals in this age group, especially in Korea. Previously, we investigated postnatal Hg exposure and its relationships with anthropometry and dietary factors in healthy Korean weaning-age infants [25]. In the present study, we investigated the exposure levels of four potentially growth-affecting toxic metals (As, Cd, Hg, and Pb) in a different population with similar characteristics. The aim of this study was to explore the relationship between growth, feeding method, diet during the weaning period, iron levels, and the heavy toxic metal burden in weaning-age Korean infants.

## 2. Materials and Methods

### 2.1. Subjects and Study Design

This study was conducted in healthy infants who visited the Pediatric Clinic of Seoul Metropolitan Government, Seoul National University, Boramae Medical Center for health examinations and iron deficiency screenings from July 2014 to June 2016. All study participants resided in Seoul, Korea and were covered by self-paid national medical insurance with no fully or partially paid medical care. Inclusion criteria for children were as follows: ranging from 8 to 23 months in age and healthy; no intake of herbal medicine, iron, or zinc supplements in the past three months; no acute febrile disease or acute gastrointestinal disease in the past two weeks; and no evidence of other acute or chronic diseases affecting growth on physical examination or in medical history. The study protocol was approved by the Boramae Hospital Institutional Review Board (16-2013-55, 16-2015-83), and written informed consent was obtained from the parents of all participants. The estimated number of subjects was greater than 186 according to sample size calculations based on our preliminary data (type I error = 0.05, power = 0.8, partial correlation coefficient between head circumference Z scores and blood As levels = −0.204).

Each infant’s weight, height, and head circumference was measured by experienced nurse, as previously described [40]. An experienced pediatrician collected birth-related, parents-related and dietary information from his/her parents through modified validated questionnaires as described previously [25]. Birth-related information included birth order, gestational age, birth weight, head circumference at birth, mother’s age at delivery, and type of delivery (Table 1). Head circumference at birth was collected only in a subset of participants, whose parents showed the records for anthropometry at birth or whose electronic medical records at birth were available. Information on the mother’s smoking status before pregnancy and during pregnancy and the father’s indoor smoking status was also collected. For participants enrolled from July 2015, the parents’ highest education level was obtained. Dietary information included the type of feeding, duration of feeding, and the dominant method of feeding during early and late infancy. Information on CF intake patterns was also collected, including the starting age for rice, red meat, and fish consumption and the recent average daily amounts of rice-based foods and red meat intake. The parents’ subjective assessment on the adequacy of CF intake was re-evaluated by the same experienced pediatrician based on the information provided on recent average rice-based food and red meat intake (Table 1).

### 2.2. Blood Tests

To diagnose iron deficiency (ID) and iron deficiency anemia (IDA), complete blood count, serum iron and ferritin concentrations, as well as total iron-binding capacity were measured from the venous blood samples of infants as previously described [25,40]. The As, Cd, Hg, and Pb levels in the same venous whole blood samples of the infants were determined using Inductively-Coupled Plasma Mass Spectrometry (Agilent 7700, Santa Clara, CA, USA) at the Green Cross Reference Laboratory (Yongin-City, Kyunggi-do, Korea) as previously described [25].

### 2.3. Definition

Anthropometry at birth and at the study time was expressed as a Z-score according to age and sex using the Anthro software (version 3.2.2., January 2011) [44]. Post-birth weight gain was expressed as the difference in Z-scores (WAZ–BWZ), which was calculated as the difference in Z-scores for weight for age (WAZ) and the Z-scores for birth weight (BWZ) at the time of the study.

The dominant feeding method was categorized as exclusively/mostly breastfeeding, mostly formula feeding, and mixed feeding. Exclusively/mostly breastfeeding and mostly formula feeding were defined as previously described [25]. The infants who were not classified as mostly breastfed or mostly formula fed were defined as have mixed feeding. The duration of breastfeeding was counted as the period of total breastfeeding (≥2 times/day).

Higher toxic metal levels were defined as those equal to or greater than median values, and lower levels as those less than median values. The upper limits of blood metal levels except As, followed those recommended by the U.S. Department of Labor, Environmental Protection Agency (EPA), and Centers for Disease Control and Prevention (CDC) of the US as follows: Cd, 5 μg/L; Hg, 5.8 μg/L; Pb, 5 μg/dL [3,15,45]. ID was defined as (1) a serum ferritin level of <12 ng/mL or (2) mean corpuscular volume of <70 fL and transferrin saturation of <16% [46]. IDA was defined as Hb < 11 g/dL plus ID.

### 2.4. Statistical Analysis

All analyses were performed using PASW software (version 20.0; SPSS Inc., Chicago, IL, USA). Normality was tested by visually inspecting normal distribution plots and histograms as well as residual plots of variables. Normally distributed variables such as Z-scores were expressed as mean ± standard deviation and were tested using the Student’s *t*-test. Non-normally distributed variables including blood toxic metal levels were expressed as the median and interquartile range (IQR) and were tested using the Mann-Whitney test or Kruskal-Wallis test. Categorical data were compared using the Pearson’s χ^2^ test. Correlations between continuous variables were assessed using either the Pearson correlation coefficient or the Spearman rank correlation coefficient. Based on the results of correlation tests and simple linear regression tests, two adjusted linear regression models for post-birth weight gain Z-scores and head circumference Z-scores were built. Explanatory variables included potential growth-affecting parameters, including blood toxic metal levels, birth weight, sociodemographic and feeding-related factors, and iron and anemia status, using a backward elimination method. In addition, two adjusted linear regression models (for As and Hg) and two logistic regression models (for Cd and Pb) were used to build four prediction models for blood toxic metal levels, based on the participants’ sociodemographic and feeding-related factors and iron status using a backward elimination method. For these analyses, the common logarithm-transformed values (base 10) of log As and Hg (ng/dL) were used because As or log As and Hg levels were not normally distributed. Thus, for As, the log of the log-change score of As levels (ng/dL), log (log As), was used to further improve the normality of the residuals. For Cd and Pb, categorical values based on median values (Supplementary Tables S1 and S2) were used instead because the transformation of measured metal levels into normally distributed values was not possible.

## 3. Results

### 3.1. Characteristics of the Study Subjects

A total of 210 term infants were included in this study, with a median age of 11.4 months (range: 8.7–22.0; IQR: 10.5–12.0), and the sex ratio was 97 male infants to 113 female infants (M/F = 0.86) (Table 1). The birthweight for age (WAZ) was −0.08 (95% CI: −0.18–0.02), and the number of underweight (WAZ < −2) and overweight infants (WAZ > 2) was 0 and 10, respectively. The anthropometric Z-scores for weight, height, and head circumference are summarized in Table 1. In terms of feeding type, 95 infants (45.2%) were exclusively or mostly breastfed until late infancy for a median time of 11.4 months (IQR: 10.5–12.0), and 66 (31.4%) were exclusively or mostly formula-fed. The median duration of complementary feeding was 5.0 months (IQR: 5.0–6.0). Rice-based porridge/cooked rice ingestion and red meat intake were perceived as adequate in 163 (71.5%) and 166 (72.8%) infants, respectively. There were 121 (57.6%) infants that were fed fish more than once per week. ID and IDA were observed in 44 (21.0%) and 18 (8.6%) infants, respectively. In terms of the related characteristics of parents, no maternal smoking history during pregnancy was identified in any of the participants. Indoor smoking by the father was identified in 46 (21.7%) participants. Among 99 participants who were evaluated from July 2015, the highest education level of the fathers and mothers were postgraduate education (17.2% and 18.2%), college or university graduate (71.7% and 71.7%), and high school graduate (11.1% and 10.1%).

### 3.2. Blood Toxic Metal Levels and Their Interrelationship

Among the 210 infants, the median blood As, Cd, Hg, and Pb levels were 1.2 μg/L (IQR 1.62–2.26), 0.05 μg/L (IQR 0.06–0.07), 0.8 μg/L (IQR 0.90–0.11), and 0.83 μg/dL (IQR 0.86–1.05), respectively (Table 2). Blood Cd, Hg, and Pb levels in all infants were lower than 5 μg/L, 5.8 μg/L, and 5 μg/dL, respectively. There was a weak but significant correlation among blood As, Cd, and Hg levels (As–Cd, r = 0.23, *p* = 0.001; As–Hg, r = 0.46, *p* < 0.001; Cd–Hg, r = 0.23, *p* < 0.001); however, blood Pb levels were not significantly correlated with any other blood toxic metal levels.

### 3.3. Association between Blood Toxic Metal Levels and Growth Parameters

Because there was a significant correlation between blood Pb levels and post-birth weight gain (r = −0.202, *p* = 0.003) or head circumference for age Z scores (HCAZ) (r = −0.190, *p* = 0.006) and blood As level and HCAZ (r = −0.159, *p* = 0.023) (Supplementary Figures S1 and S2), we performed multiple linear regressions with growth-related parameters as dependent variables (Table 3). There was still a significant correlation between weight gain after birth and blood Pb levels (B = −0.238, *p* = 0.003) after adjustment for other potential growth-related parameters. The current head circumference was also significantly correlated with blood Pb levels (B = −0.213, *p* = 0.007) and blood As levels (B = −0.053, *p* = 0.020) after adjustment for other potential growth-related parameters.

### 3.4. The Relationship between Feeding and Dietary Factors and Blood Toxic Metal Levels

Blood Cd, Hg, and Pb levels were significantly higher in the infants who were mostly breastfed until late infancy compared to those who were mostly formula-fed (Cd: *p* = 0.001; Hg and Pb: *p* < 0.001; Table 4). The duration of breastfeeding was also significantly correlated with each blood metal level (As: r = 0.181, *p* = 0.009; Cd: r = 0.273, *p* < 0.001; Hg: r = 0.394, *p* < 0.001; Pb: r = 0.427, *p* < 0.001).

There were significant associations between some dietary patterns during weaning and certain blood metal levels. Blood As levels were significantly higher in the infants with perceived adequacy of rice-based CF intake (*p* = 0.013). In contrast, blood Pb levels were significantly lower in those same infants (*p* = 0.001). Blood Pb levels were also significantly lower in the infants with perceived adequacy of red meat intake (*p* = 0.004). Blood As and Hg levels were significantly higher in the infants with regular fish intake (*p* < 0.001, *p* = 0.006, respectively).

In adjusted linear regression models (Table 5) and adjusted logistic regression models (Table 6), the duration of breastfeeding was significantly associated with blood log (log As) (B = 0.004, *p* = 0.002), log Hg (B = 0.028, *p* < 0.001), higher Cd levels (adjusted odds ratio, a-OR: 1.1, *p* = 0.019), and higher Pb levels (a-OR: 1.3, *p* < 0.001). Perceived adequacy of rice-based CF intake and duration of CF intake were significantly associated with blood log (log As) (B = 0.022, *p* = 0.044; B = 0.006, *p* = 0.017, respectively). Fish intake was significantly associated with blood log (log As) (B = 0.036, *p* < 0.001) and log Hg levels (B = 0.036, *p* < 0.001).

### 3.5. The Relationship between Iron Deficiency and Blood Toxic Metal Levels

Blood Cd and Pb levels were significantly higher in infants with ID compared to those without ID (Cd: *p* = 0.029; Pb: *p* < 0.001, Table 4). Blood Pb levels, but not blood Cd levels, were also significantly higher in infants with IDA compared to those without IDA (*p* < 0.001). Other blood metal levels were not correlated with ID or IDA.

In adjusted logistic regression models (Table 6), ID tended to be associated with higher blood Cd levels (a-OR: 2.3, *p* = 0.071), and IDA was significantly associated with higher blood Pb levels (a-OR: 9.9, *p* = 0.030).

### 3.6. The Relationship between Other Sociodemographic Factors and Blood Toxic Metal Levels

There were significant positive correlations between age and blood As, Cd, and Hg levels (As: r = 0.282, *p* < 0.001; Cd: r = 0.278, *p* < 0.001; Hg: r = 0.278, *p* < 0.001). However, age was not significantly correlated with blood Pb levels. There was a tendency of a weak correlation between gestational age and blood Hg or Pb levels (Hg: r = 0.135, *p* = 0.051; Pb: r = 0.118, *p* = 0.088), although it was not significant. First birth order was significantly associated with higher blood Hg levels (0.91 (0.61, 1.26) vs. 0.66 (0.47, 1.15), *p* = 0.026). Mother’s age at delivery (≥30 years) was significantly associated with higher As levels (0.95 (0.5, 1.63) vs. 1.3 (0.83 vs. 2.08), *p* = 0.041). The father’s indoor smoking was significantly associated with higher blood Pb levels (0.75 (0.47, 1.15) vs. 1.04 (0.72, 1.52), *p* = 0.002) (Supplemental Table S2). The highest education level of mothers (*n* = 99) was significantly associated with lower blood Hg levels (a college education or greater versus a high school graduate; 0.72 (0.5, 1.12) vs. 1.76 (0.65, 2.01), *p* = 0.006). Sex, method of birth, and maternal smoking before pregnancy were not significantly correlated with any blood metal levels.

In adjusted linear regression models (Table 5) or adjusted logistic regression models (Table 6), monthly age was significantly associated with higher blood Cd levels (a-OR: 1.7, *p* = 0.001) and marginally associated with blood log Hg levels (B = 0.021, *p* = 0.097). Male children tended to have higher Cd levels (a-OR: 1.8, *p* = 0.059). The mother’s age at delivery (≥30 years) was significantly associated with log (log As) levels (B = 0.029, *p* = 0.044). The father’s indoor smoking was significantly associated with higher Pb levels (a-OR: 2.7, *p* = 0.017). Other demographic parameters were not significantly associated with any blood metal levels.

## 4. Discussion

This study shows that Cd, Hg, and Pb exposure in healthy Korean weaning-age infants residing in Seoul is usually below the upper limit suggested by the EPA and CDC of the U.S. [3,15,45]. Median Cd and Hg levels were distinctively lower in our subjects than in a small subset of Korean newborns or school-aged children, and Pb levels were slightly lower in our subjects than in Korean or western school-aged children [47,48,49]. The globally accepted upper normal limit blood level for As has not yet been established [50]. However, according to the 12 μg/L upper limit suggested by the Mayo Clinic in the U.S. [51], all infants had acceptable blood As levels. Still, according to the 1.0 μg/L upper limit from the Agency for Toxic Substances and Disease Registry [3], 66.7% of our subjects had higher levels than desired. Although few studies investigating blood As levels among weaning-age infants are available in the literature, the geometric mean As value of the infants in our study was similar to the cord blood As levels of Nepal infants; blood As levels are distinctively lower than that of adults from high-risk areas such as Bangladesh, and distinctively higher than that of Western adults and children [52,53,54,55]. This matches the results from a previous Korean national study showing that urine As levels in Korean adults were similar to those from eastern Asia but much higher than those in western adults [56,57]. However, because blood and urine in Koreans may often contain a considerable amount of organic As through seafood ingestion (which is regarded as far less toxic than inorganic As), the differences in blood As levels between the infants in our study and the infants in western populations may not equal the differences in toxicity between them [37,56].

Among the four toxic metals, only As and Pb levels were negatively associated with anthropometry of Korean weaning-age infants in adjusted linear regression models. As was only associated with current head circumference, whereas Pb was associated with both post-birth increase of weight and current head circumference. A direct comparison of the degree of the adverse influence of As and Pb on post-birth growth through blood levels might not be possible. However, Pb seemed to influence growth restriction factors to a greater degree in an adjusted linear model for current head circumference, although the range of Pb levels was lower in our subjects than in those from previous growth-related studies [8,12,16]. To our knowledge, this is a unique result showing that low level postnatal Pb exposure, mostly <3 µg/dL, may be associated with restricting post-birth growth, including head size increase of children during infancy. This study is also noteworthy because a significant inverse association between low to modest As exposure and post-birth anthropometry was observed among subjects who did not reside in previously known high-risk areas with As-contaminated water exceeding WHO standards [29,58]. Compared to the other three toxic metals, investigations on the association of low levels of prenatal or postnatal As exposure and post-birth anthropometry in children are extremely scarce. In addition, this is one of the first reports demonstrating the inverse association between low As and low Pb levels and post-birth growth within the same population in the weaning period, in which potential growth-affecting factors including ID or IDA and feeding type or diet adequacy were adjusted.

The method by which As restricts growth is unclear. Endocrine-disrupting properties leading to insulin-like growth factor 1 (IGF-1) suppression have been suggested as a mechanism of growth impairment [59]. Our study cannot explain the reason why As was associated with only current head circumference and not post-birth weight gain. It may be associated with the toxic nature of low As exposure itself and/or a specific exposure period during childhood. We cannot exclude the possibility that a smaller head circumference at birth associated with intrauterine As exposure may considerably contribute to the lower head circumference in this study, because brain growth is particularly vulnerable to intrauterine insult [60]. Our study might be limited in its ability to prove an independent association between postnatal As exposure and current head circumference because the cord-blood As levels were not investigated, although mother’s age at delivery was adjusted, which might be associated with cord-blood As levels. In addition, as the current head circumference of our infants was mostly within normal range, the clinical significance of the observed inverse association between As and growth may need to be determined through further functional study. In a previous Nepal cohort, cord blood As levels, similar to ours, were associated with adverse neurodevelopment at birth, but not at 36 months of age [54]. Although many studies used the urinary As level as a biomarker for exposure [61], blood As has the advantage of reflecting the As burden in tissue compartments [55]. In general, blood As concentrations have been considered to reflect only recent exposure. However, considering that the feeding patterns and diet of weaning-age infants are usually repetitive once established and maintained long term, blood As levels can reflect long-term steady-state exposure [55]. We did not find an independent association between low-level post-birth Cd or Hg exposure and post-birth anthropometry, which is consistent with the results of a few previous studies [13,25]. It may be associated, at least in part, with much lower Cd levels in our subjects compared to newborns or older children in Korea or western countries [47,49].

Feeding type and breastfeeding duration were significantly correlated with all four metal levels in our study, which was consistent with the results of previous studies [29]. The relatively slow growth of breastfed infants compared with formula-fed infants during the first few years of life is well documented [62]. Consequently, it is important to adjust the type of feeding or amount and duration of breastfeeding to document the association between toxic metals and infant growth. In this study, although post-birth Z-scores were negatively correlated with the duration of breastfeeding, As and Pb levels were chosen as final explanatory variables, rather than feeding type or duration of breastfeeding, in the adjusted regression models for anthropometry, which was contrary to the case of Hg levels in our previous study [25]. Therefore, breastfeeding should be encouraged, and As and Pb exposure should be reduced as much as possible by controlling the diet and environment of breastfeeding mothers and their babies. In fact, in the case of As, there are also reports that the inorganic As secretion of breastmilk is decreased by increased methylation in lactating mothers; thus, the As concentration in breast milk is very low even in contaminated areas, unlike that of Hg [29,63]. Pb levels seemed more affected by IDA than by CF type in our subjects. Given that prolonged breastfeeding is also a well-documented risk factor for ID or IDA [64,65], prevention of ID or IDA can be one of the most important precautions for reducing toxic metal exposure in breastfed infants. In this study, ID was also found to be associated with higher Cd levels, which was consistent with the results from previous studies on adults and children [43,66].

There was a significant correlation between the adequacy of specific food intake and specific toxic metal levels in weaning-age infants, similar to Korean adults and older children [37,38]. Although the amount of food intake was not precisely quantified in this study, the perceived adequacy of rice-based food intake was significantly associated with blood As levels and it tended to be associated with blood Cd levels as well. Infants with fish intake more than once per week also had increased blood As and Hg levels. In general, while As in both rice and breast milk is regarded as highly toxic inorganic As, seafood contains organic As, which does not induce substantial damage to the body because it is rapidly and completely excreted in the urine [63]. However, attention to the As exposure through seafood intake is also required, as recent studies showed increased levels of dimethylarsinic acid (DMA) in the urine of adult populations after seafood intake, which is far more toxic than organic As [67].

There are some limitations to this study. It was a single-center, cross-sectional study with a modest number of infants. Toxic metal levels were measured only once. Precise quantification of dietary intake was not performed. While blood is the preferred biomarker for Pb and Hg exposure, urine is the usual biomarker for As and Cd exposure in many studies. Finally, this study did not thoroughly adjust for environmental, genetic, social, and economic factors, which could potentially affect growth. Nevertheless, based on information about the medical insurance status, detailed addresses of the residence area, and smoking history of mothers, it is possible to assume that our subjects may likely not be at risk for a poor nutritional status or environmental exposure to toxic metals. In half of the study participants, the parents’ highest education level was available, which showed that the proportion of highest education levels were higher in our study population than in the general Korean population with similar ages.

## 5. Conclusions

To our knowledge, this is a unique study in that it measured toxic metal levels of weaning-age infants using blood levels as biomarkers and obtained an integrated picture of the close association between anthropometry, breastfeeding, CF, and ID or IDA of infancy in the same population. Although Cd, Hg, and Pb exposure in healthy Korean weaning-age infants residing in Seoul is usually below the officially suggested upper limit, the As and Hg levels were found to be higher than those in western populations, as in Korean adults. Among the four toxic metals, As and Pb exposure showed significant negative associations with anthropometry in weaning-age infants. The toxic metal exposure levels were also significantly associated with the type of feeding or duration of breastfeeding, perceived adequacy of a specific diet, and iron and anemia status. Although levels of toxic metals may not usually be high in this population, individual exposure risk may need to be assessed after considering the type of feeding or intake of complementary foods and the iron/anemia status during evaluation of growth status. In addition, meticulous parental education for prevention of ID and IDA as well as efforts to maintain the appropriate iron status through screening or preventive iron intake are necessary.

## Figures and Tables

**Table 1:** Demographic, anthropometric, and diet-related parameters and iron status of 210 infants.

Characteristics	Value
Age, *months* ^a^	11.4 (10.5, 12.0)
Sex, *n*	
Male	97
Female	113
Birth order	
First	157
≥Second	53
Mother’s age at delivery	
<30 years	22
≥30 years	188
Type of birth, *n*	
Vaginal delivery	148
Surgical delivery	62
Gestational age, *weeks* ^a^	39.0 (38.0, 40.0)
Anthropometry, *Z-score* ^b^	
Birthweight, kg ^a^	3.24 (3.02, 3.48)
Birthweight	−0.08 ± 0.73
Weight for age	0.65 ± 0.82
WAZ–BWZ	0.73 ± 0.89
Height for age	0.53 ± 1.09
Head circumference at birth, cm ^c^	34.0 (33.5, 35.0)
Head circumference at birth ^c^	0.08 ± 0.87
Head circumference for age	0.44 ± 0.83
Post-birth growth of head circumference ^c,d^	0.29 ± 1.12
Feeding type, *n*	
Exclusively/mostly breastfed	95
Mixed fed	49
Mostly formula fed	66
Duration of breastfeeding, *months* ^a^	10.0 (5.0, 11.4)
Duration of CF intake, *months* ^a^	5.0 (5.0, 6.0)
Adequacy of rice-based food intake, *n*	
Adequate	163
Poor	47
Duration of red meat intake, *months* ^a^	5.0 (4.0, 6.0)
Adequacy of red meat intake, *n*	
Adequate	166
Poor	44
Regular fish intake, *n*	
Presence	121
Absence	89
Duration of fish intake, *months* ^a^	1.0 (0.0, 3.0)
Iron status, *n*	
Deficiency	44
No deficiency	166
Iron deficiency anemia	18
Mother’s smoking before pregnancy	
No	202
Yes	8
Father’s indoor smoking	
No	164
Yes	46

^a^ The values are presented as the median and interquartile range; ^b^ The values are presented as the mean and standard deviation (95% confidence interval, CI); ^c^ The measurements were evaluated in 112 infants whose parents provided their babies’ records for head circumference at birth or whose electronic medical records at birth were available; ^d^ The values were calculated as the difference in the current head circumference Z scores minus the head circumference Z scores at birth. WAZ–BWZ: The difference of the weight for age Z scores at the time of the study and birth weight Z scores; CF: Complementary feeding.

**Table 2:** Blood toxic metal levels in 210 infants.

	Geometric Mean	Percentiles					Maximum
	(95% CI)	10	25	50	75	90	Value
Blood metal level							
As (μg/L)	1.94 (1.62, 2.26)	0.5	0.8	1.2	2.0	4.4	11.9
Cd (μg/L)	0.067 (0.06, 0.073)	0.02	0.04	0.05	0.09	0.11	0.4
Hg (μg/L)	0.99 (0.90, 1.08)	0.34	0.56	0.8	1.24	1.8	4.2
Pb (μg/dL)	0.96 (0.86, 1.05)	0.12	0.52	0.83	1.23	1.82	3.5

**Table 3:** Adjusted linear regression models for growth parameters in 210 infants according to possible growth-related factors including toxic metal levels.

Dependent Variables	Independent Variables	Unadjusted		Adjusted	
		B (SE)	*p*-Value	B (SE)	*p*-Value
WAZ–BWZ	BWZ	−0.601 (0.073)	<0.001	−0.586 (0.073)	<0.001
	Iron deficiency	0.217 (0.150)	0.150	0.223 (0.131)	0.09
	Blood Pb levels	−0.202 (0.089)	0.024	−0.238 (0.078)	0.003
HCAZ	BWZ	0.352 (0.075)	<0.001	0.358 (0.074)	<0.001
	Blood Pb levels	−0.206 (0.083)	0.014	−0.213 (0.078)	0.007
	Blood As levels	−0.057 (0.024)	0.018	−0.053 (0.022)	0.020

WAZ–BWZ: The difference of the weight for age Z scores at the time of the study and birth weight Z scores; HCAZ: head circumference for age Z scores.

**Table 4:** Blood toxic metal levels according to dietary factors and iron status.

Parameters	As ^a^ (μg/L)	Cd ^a^ (μg/L)	Hg ^a^ (μg/L)	Pb ^a^ (μg/dL)
Feeding type				
Exclusively/mostly breastfed	1.4 (1.0, 2.1)	0.06 (0.05, 0.10)	1.1 (0.7, 1.6)	1.12 (0.77, 1.63)
Mixed fed	1.0 (0.7, 1.7)	0.04 (0.03, 0.08)	0.8 (0.5, 1.1)	0.81 (0.51, 1.11)
Mostly formula fed	1.1 (0.6, 2.1)	0.05 (0.03, 0.07)	0.7 (0.4, 1.0)	0.62 (0.39, 0.82)
*p*-Value ^b^	0.065	0.001	<0.001	<0.001
Adequacy of rice-based food intake				
Adequate	1.4 (0.9, 2.2)	0.06 (0.04, 0.10)	0.8 (0.6, 1.3)	0.74 (0.51, 1.12)
Poor	1.0 (0.6, 1.7)	0.05 (0.03, 0.07)	0.7 (0.5, 1.2)	1.06 (0.77, 1.58)
*p*-Value	0.013	0.079	0.219	0.001
Adequacy of red meat intake				
Adequate	1.3 (0.9, 2.1)	0.06 (0.04, 0.10)	0.8 (0.6, 1.3)	0.75 (0.51, 1.14)
Poor	1.0 (0.7, 1.9)	0.05, 0.03, 0.07)	0.8 (0.5, 1.2)	1.04 (0.77, 1.53)
*p*-Value	0.069	0.236	0.435	0.004
Regular fish intake				
Presence	1.5 (0.9, 2.9)	0.05 (0.04, 0.10)	0.9 (0.7, 1.3)	0.74 (0.51, 1.20)
Absence	1.0 (0.7, 1.6)	0.05 (0.04, 0.08)	0.7 (0.5, 1.2)	0.97 (0.59, 1.27)
*p*-Value	<0.001	0.678	0.006	0.063
Iron Status				
Deficiency	1.4 (0.8, 2.0)	0.07 (0.05, 0.10)	1.0 (0.6, 1.4)	1.24 (0.84, 1.64)
No deficiency	1.2 (0.8, 2.0)	0.05 (0.03, 0.08)	0.8 (0.5, 1.2)	0.75 (0.51, 1.10)
*p*-Value	0.752	0.029	0.134	<0.001
Iron deficiency anemia				
Presence	1.5 (1.0, 2.0)	0.06 (0.05, 0.08)	0.9 (0.6, 1.5)	1.44 (1.14, 1.80)
Absence	1.2 (0.8, 2.0)	0.05 (0.04, 0.01)	0.8 (0.6, 1.2)	0.79 (0.51, 1.14)
*p*-Value	0.279	0.419	0.390	<0.001

^a^ The values are presented as the median and interquartile range; ^b^ The values were calculated using the Kruskal-Wallis test between mostly breastfed infants and mostly formula fed infants. Other *p*-values were calculated using the Mann Whitney test.

**Table 5:** Simple and multiple linear regression analysis of Log (log As) and Log Hg levels in 210 infants according to demographics, dietary parameters, and iron status.

Variables	Unadjusted		Adjusted	
	B (SE)	*p*-Value	B (SE)	*p*-Value
Log (log As)				
Duration of breastfeeding	0.003 (0.001)	0.008	0.004 (0.001)	0.002
Duration of CF	0.009 (0.003)	0.001	0.006 (0.003)	0.017
Adequate rice-based food intake	0.031 (0.011)	0.007	0.022 (0.011)	0.044
Regular fish intake	0.038 (0.009)	<0.001	0.036 (0.009)	<0.001
Mother’s age at delivery (≥30 years)	0.035 (0.015)	0.026	0.029 (0.014)	0.044
Log Hg				
Monthly age	0.044 (0.013)	0.001	0.021 (0.012)	0.097
Duration of breastfeeding	0.028 (0.005)	<0.001	0.028 (0.005)	<0.001
Duration of fish intake	0.033 (0.010)	0.002	0.036 (0.010)	<0.001

**Table 6:** Simple and multiple logistic regression analysis of Cd and Pb levels in 210 infants according to demographics, dietary parameters, and iron status.

Variables	Unadjusted Odds Ratio	95% CI	*p*-Value	Adjusted Odds Ratio	95% CI	*p*-Value
Cd						
Monthly age	1.7	1.3, 2.3	<0.001	1.7	1.3, 2.4	0.001
Male sex	1.9	1.1, 3.4	0.028	1.8	1.0, 3.4	0.059
Duration of breastfeeding	1.2	1.1, 1.3	<0.001	1.1	1.0, 1.2	0.019
Iron deficiency	3.0	1.3, 7.0	0.008	2.3	0.9, 5.9	0.071
Pb						
Duration of breastfeeding	1.3	1.2, 1.4	<0.001	1.3	1.2, 1.4	<0.001
Iron deficiency anemia	19.3	2.5, 147.7	0.004	9.9	1.3, 78.1	0.030
Father’s indoor smoking	2.5	1.2, 5.0	0.013	2.7	1.2, 6.0	0.017

## Supplementary Materials

The following are available online at www.mdpi.com/1660-4601/14/4/388/s1, Figure S1: Correlation between blood lead levels and post-birth weight gain (WAZ–BWZ) (a), and head circumference for age Z scores (HCAZ) (b), Figure S2: Correlation between blood arsenic levels and head circumference for age Z scores (HCAZ), Table S1: Demographic, anthropometric, and diet-related parameters and iron status in two categories of infants according to the median values of blood cadmium levels, Table S2: Demographic, anthropometric, and diet-related parameters and iron status in two categories of infants according to the median values of blood lead levels.
