# Supplementary material for: Low-Level Toxic Metal Exposure in Healthy Weaning-Age Infants: Association with Growth, Dietary Intake, and Iron Deficiency
Source: Int J Environ Res Public Health. 2017 Apr 6;14(4):388. doi: 10.3390/ijerph14040388 (PMC5409589; doi:10.3390/ijerph14040388)
Supplement: Supplementary file 1 [file ijerph-14-00388-s001.pdf]

Low-level toxic metal exposure in healthy weaning-age infants: association with growth, dietary intake, and iron deficiency

Jungil Choi, Ju Young Chang, Jeana Hong, Sue Shin, Jeong Su Park, Sohee Oh

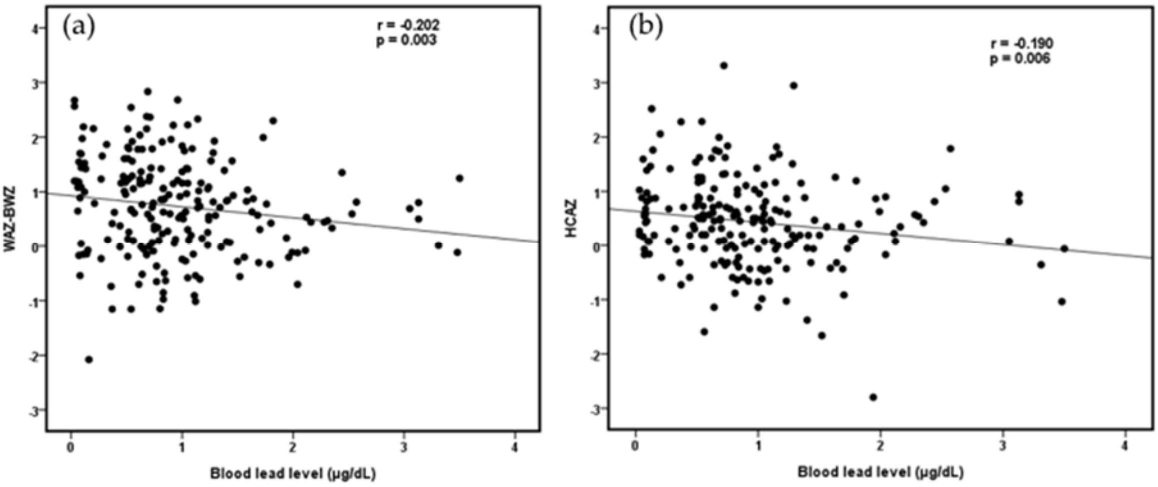

Figure S1. Correlation between blood lead levels and post-birth weight gain (WAZ–BWZ) (**a**), and head circumference for age Z scores (HCAZ) (**b**).

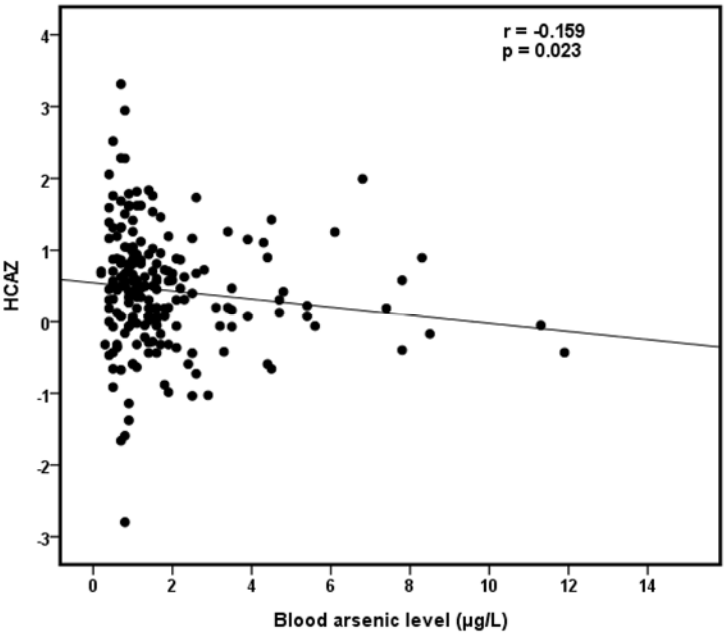

Figure S2. Correlation between blood arsenic levels and head circumference for age Z scores (HCAZ).

Table S1. Demographic, anthropometric, and diet-related parameters and iron status in two categories of infants according to the median values of blood cadmium levels.

Characteristics	Blood cadmium level (μg/L)			*p*
	<0.05 (n=75)	≥0.05 (n=135)	median (IQR)	
Age, *months* [a]	10.9 (10.3, 11.7)	11.5 (10.9, 12.3)		<0.001
Sex, *n*				
Male	27	70	0.06 (0.04, 0.10)	0.027
Female	48	65	0.05 (0.03, 0.09)	
Birth order				
First	56	101	0.05 (0.04, 0.09)	0.979
≥Second	19	34	0.06 (0.04, 0.10)	
Mother's age at delivery				
< 30 years	7	15	0.05 (0.02, 0.10)	0.687
≥30 years	68	120	0.06 (0.04, 0.09)	
Gestational age, *weeks* [a]	39.0 (38.2, 40.0)	39.0 (38.0, 40.0)		0.161
Type of birth, *n*				
Vaginal delivery	51	97	0.05 (0.04, 0.09)	0.558
Surgical delivery	24	38	0.06 (0.04, 0.09)	
Anthropometry, *Z-score* [b]				
birthweight, kg [a]	3.24 (3.0, 3.54)	3.24 (3.02, 3.47)		0.224
birthweight	0.129±0.820	-0.130±0.676		0.199
weight for age	0.665±0.841	0.648±0.816		0.891
WAZ - BWZ	0.652±0.880	0.789±0.894		0.321
height for age	0.513±1.012	0.545±1.141		0.321
HC at birth, cm[a,c]	34.0 (33.0, 35.0)	34.0 (33.5, 35.0)		0.330
HC at birth[c]	-0.04±0.85	0.13±0.92		0.366
HCAZ	0.465±0.891	0.420±0.790		0.714
Post-birth growth of HC[c,d]	0.33±1.61	0.27±0.13		0.770
Feeding type, *n*				
exclusively/mostly breastfed	17	78	0.06 (0.05, 0.10)	<0.001
mixed fed	26	23	0.04 (0.03, 0.08)	
mostly formula fed	32	34	0.05 (0.03, 0.07)	
Duration of breastfeeding, *months* [a]	6.0 (3.0, 10.3)	10.4 (6.0, 11.7)		<0.001
Duration of CF intake, *months* [a]	5.0 (4.0, 6.0)	6.0 (5.0, 7.0)		0.030
Adequacy of rice-based food intake, n				
Adequate	55	108	0.06 (0.04, 0.10)	0.267
Poor	20	27	0.05 (0.03, 0.07)	
Duration of red meat intake, *months* [a]	5.0 (3.0, 6.0)	5.0 (4.0, 6.0)		0.001
Adequacy of red meat intake, *n*				
Adequate	57	109	0.06 (0.04, 0.10)	0.419

Poor	18	26	0.05, 0.03, 0.07)	
Regular fish intake, *n*				
Presence	43	78	0.05 (0.04, 0.10)	0.950
Absence	32	57	0.05 (0.04, 0.08)	
Duration of fish intake, *months* [a]	1.0 (0.0, 3.0)	1.0 (0.0, 3.0)		0.459
Iron status, *n*				
Deficiency	8	36	0.07 (0.05, 0.10)	0.006
No deficiency	67	99	0.05 (0.03, 0.08)	
Iron deficiency anemia	4	14	0.06 (0.05, 0.08)	0.212
Mother's smoking before pregnancy				
No	72	130	0.06 (0.02, 0.08)	0.914
Yes	3	5	0.05 (0.04, 0.10)	
Father's indoor smoking				
No	55	109	0.05 (0.03, 0.07)	0.214
Yes	20	26	0.06 (0.04, 0.10)	

P values were calculated using the chi-square test unless stated otherwise. [a]The values are presented as the median and interquartile range. The comparisons according to blood Cd levels were performed using the Mann-Whitney's test. [b]The values are presented as the mean and standard deviation (95% confidence interval). The comparisons according to blood Cd levels were performed using the independent samples t test. [c]The measurements were evaluated in 112 infants whose parents provided their babies' records for head circumference at birth or whose electronic medical records at birth were available; [d]The values were calculated as the difference in the current head circumference Z scores minus the head circumference Z scores at birth. WAZ-BWZ: The difference of the weight percentiles between the time of the study and birth; HCAZ: head circumference for age Z scores

Table S2. Demographic, anthropometric, and diet-related parameters and iron status in two categories of infants according to the median values of blood lead levels.

**Characteristics**	**Blood lead level (μg/dL)**			***p***
	<0.83 (n=75)	≥0.83 (n=135)	median (IQR)	
Age, *months* [a]	11.2 (10.4, 12.0)	11.4 (10.7, 12.0)		0.203
Sex, *n*				
Male	44	53	0.84 (0.56,1.27)	0.322
Female	59	54	0.73 (0.50, 1.20)	
Birth order				
First	79	78	0.82 (0.52, 1.16)	0.526
≥Second	24	29	0.89 (0.63, 1.66)	
Mother's age at delivery				
< 30 years	10	12	0.95 (0.32, 1.16)	0.722
≥30 years	93	95	0.83 (0.54, 1.24)	
Gestational age, *weeks* [a]	39.0 (38.0, 40.0)	39.3 (38.1, 40.0)		0.094
Type of birth, *n*				
Vaginal delivery	70	78	0.85 (0.54, 1.24)	0.433
Surgical delivery	33	29	0.73 (0.50, 1.14)	
Anthropometry, *Z-scores* [b]				
Birthweight, kg [a]	3.24 (3.0, 3.48)	3.24 (3.04, 3.49)		0.403
Birthweight for age	-0.084 0.796	-0.075 0.669		0.925
Weight for age	0.774±0.860	0.540±0.771		0.039
WAZ - BWZ	0.858±0.962	0.614±0.793		0.047
Height for age	0.626±1.060	0.444±1.125		0.227
HC at birth, cm [a,c]	34.0 (33.5, 35.0)	34.3 (33.5, 35.0)		0.871
HC at birth [c]	0.04±0.95	0.10±0.86		0.751
HCAZ	0.609±0.813	0.270±0.806		0.003
Post-birth growth of HC [c,d]	0.509±1.19	0.096±1.24		0.064
Feeding type, *n*				
exclusively/mostly breastfed	27	68	1.12 (0.77, 1.63)	<0.001
mixed fed	26	23	0.81 (0.51, 1.11)	
mostly formula fed	50	16	0.62 (0.39, 0.82)	
Duration of breastfeeding, *months* [a]	6.0 (3.0, 10.1)	10.9 (9.2, 11.5)		<0.001
Duration of CF intake, *months* [a]	6.0 (5.0, 6.0)	5.0 (5.0, 6.0)		0.459
Adequacy of rice-based food intake, n				
Adequate	87	76	0.74 (0.51, 1.12)	0.020
Poor	16	31	1.06 (0.77, 1.58)	
Duration of red meat intake, *months* [a]	5.0 (4.0, 6.0)	5.0 (4.0, 6.0)		0.152
Adequacy of red meat intake, *n*				
Adequate	88	78	0.75 (0.51, 1.14)	0.026

Poor	15	29	1.04 (0.77, 1.53)	
Regular fish intake, *n*				
Presence	66	55	0.74 (0.51, 1.20)	0.063
Absence	37	52	0.97 (0.59, 1.27)	
Duration of fish intake, *months* [a]	2.0 (0.0, 4.0)	0.0 (0.0, 2.0)		
Iron status, *n*				
Deficiency	10	34	1.24 (0.84, 1.64)	<0.001
No deficiency	93	73	0.75 (0.51, 1.10)	
Iron deficiency anemia	1	17	1.44 (1.14, 1.80)	<0.001
Mother's smoking before pregnancy				
No	98	104	0.76 (0.64, 1.12)	0.438
Yes	5	3	0.83 (0.52, 1.24)	
Father's indoor smoking				
No	88	15	0.75 (0.47, 1.15)	0.012
Yes	76	31	1.04 (0.72, 1.52)	

P values were calculated using the chi-square test unless stated otherwise. [a] The values are presented as the median and interquartile range. The comparisons according to blood Cd levels were performed using the Mann-Whitney's test. [b]The values are presented as the mean and standard deviation (95% confidence interval). The comparisons according to blood Cd levels were performed using the independent samples t test. [c]The measurements were evaluated in 112 infants whose parents provided their babies' records for head circumference at birth or whose electronic medical records at birth were available; [d]The values were calculated as the difference in the current head circumference Z scores minus the head circumference Z scores at birth. WAZ-BWZ: The difference of the weight percentiles between the time of the study and birth; HCAZ: head circumference for age Z scores

© 2017 by the authors. Licensee MDPI, Basel, Switzerland. This article is an open access article distributed under the terms and conditions of the Creative Commons Attribution (CC BY) license (http://creativecommons.org/licenses/by/4.0/).
